# Transient Loss of Consciousness Associated With Severe Hypocalcemia and QT Prolongation Due to Primary Hypoparathyroidism in an Adolescent Girl

**DOI:** 10.7759/cureus.34352

**Published:** 2023-01-29

**Authors:** Masafumi Miyao, Yoshihiro Aoki, Naoto Mizushiro, Reiko Kitazawa, Chizuko Nakamura

**Affiliations:** 1 Department of Pediatrics, Aizawa Hospital, Matsumoto, JPN; 2 Department of Emergency and Critical Care Medicine, Aizawa Hospital, Matsumoto, JPN; 3 Coordination Office for Emergency Medicine and International Response, Acute and Critical Care Center, Nagasaki University Hospital, Nagasaki, JPN; 4 Department of Pediatrics, Shinshu University School of Medicine, Matsumoto, JPN

**Keywords:** hypoparathyroidism, qt prolongation, pediatrics, adolescent, calcium, syncope, seizure, hypocalcemia, emergency medicine, electrocardiography

## Abstract

As hypocalcemia is uncommon, serum calcium levels are not routinely measured in many emergency medicine clinics. We report a case of an adolescent girl with a transient loss of consciousness due to hypocalcemia. A 13-year-old healthy girl had a syncopal episode complicated with numbness in the extremities. On admission, she was fully conscious, but hypocalcemia and QT prolongation were noted. After careful consideration of the possible etiologies, the patient was diagnosed with acquired QT prolongation due to primary hypoparathyroidism. The patient’s serum calcium levels were controlled by activated vitamin D and calcium supplementation. Primary hypoparathyroidism-associated hypocalcemia can cause QT prolongation and neurological complications, even in previously healthy adolescents.

## Introduction

Hypocalcemia causes various symptoms. Its leading causes are hypoparathyroidism, vitamin D deficiency, and abnormal magnesium metabolism [1]. Hypoparathyroidism can be divided into primary hypoparathyroidism, caused by endogenous disorders of the parathyroid glands, and secondary hypoparathyroidism, caused by resection or destruction of the parathyroid glands [2]. Primary hypoparathyroidism is uncommon, and most cases are secondary due to factors such as thyroid surgery.

Severe hypocalcemia can cause seizures [2]. Seizures do not always present with jerking movements of the arms and legs but can also present as loss of consciousness; therefore, hypocalcemia should be considered in children presenting to the emergency department with unexplained transient loss of consciousness. Hypocalcemia can also cause ventricular arrhythmias with syncope due to QT prolongation. Because primary hypocalcemia is rare, serum calcium may not be measured routinely as part of initial biochemical tests in patients with transient loss of consciousness if the patient is fully conscious on admission, especially in resource-limited settings.

Few cases of hypoparathyroidism have been reported in which the patient presented with cardiac syncope due to prolonged QT interval. Herein, we report a case of an adolescent girl who presented to our emergency department with a transient loss of consciousness due to hypocalcemia caused by primary hypoparathyroidism.

## Case presentation

A 13-year-old girl was transported to our hospital due to an unexplained syncopal episode. The patient had been born preterm (36 weeks and 0 days) with low birth weight (2,338 g) and had experienced mild neonatal asphyxia. No abnormalities in her growth and development had been noted. She had not undergone anterior neck surgery. There was no family history of hypocalcemia, cardiac arrhythmia, or epilepsy.

Periodic school health examinations, including a routine electrocardiogram (ECG) performed at 12-13 years of age, had not revealed any notable abnormalities. An ECG performed during the school examinations two months before the visit had shown a normal corrected QT interval (QTc, 0.437 s). On the day of admission to our medical center, the patient experienced a sudden loss of consciousness while standing in the kitchen and fell. Her father thought that she might have cardiopulmonary arrest and performed chest compressions. Her consciousness recovered immediately, and she was transported to the emergency department by ambulance.

On admission, the patient’s Glasgow Coma Scale (GCS) score was 15. Her vital signs were: body temperature, 36.4℃; heart rate, 99 beats/min; blood pressure, 118/57 mmHg; respiratory rate 21 breaths/min; and oxygen saturation, 96% breathing room air. The physical examination findings were unremarkable: no dysmorphic facial features, facial or limb paralysis, seizure, Trousseau’s signs, or costovertebral angle tenderness were noted. The patient complained of numbness in her extremities.

**Figure 1:**
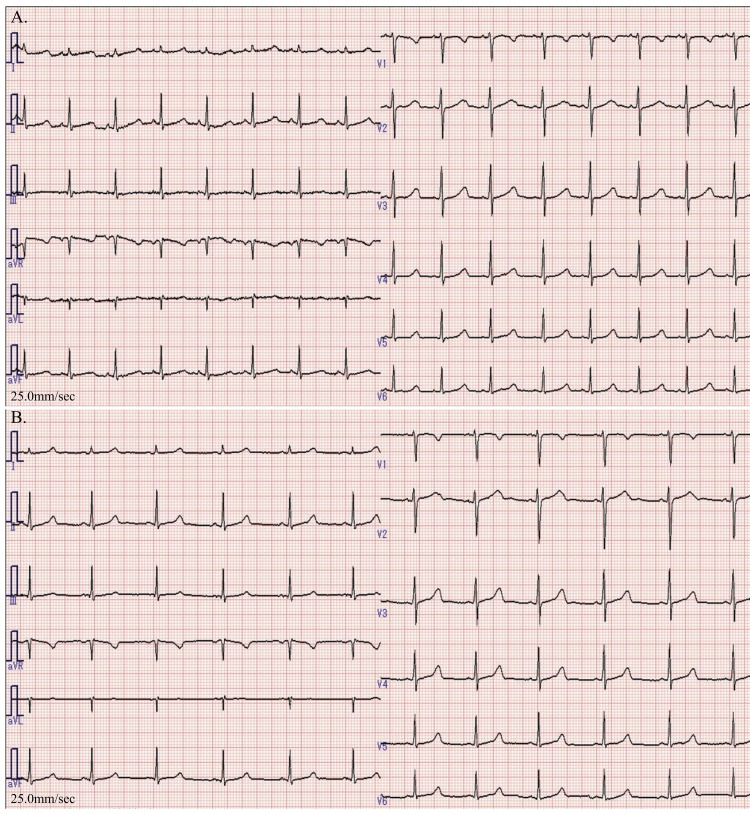
Electrocardiogram (ECG) findings on admission (A) and on hospital day 2 (B) (A) An ECG performed on admission shows sinus rhythm with marked QT prolongation (QTc = 0.524 s). (B) An ECG performed on hospital day 2 shows sinus rhythm with a shortened QT interval (QTc = 0.465 s). QTc: corrected QT interval

**Figure 2:**
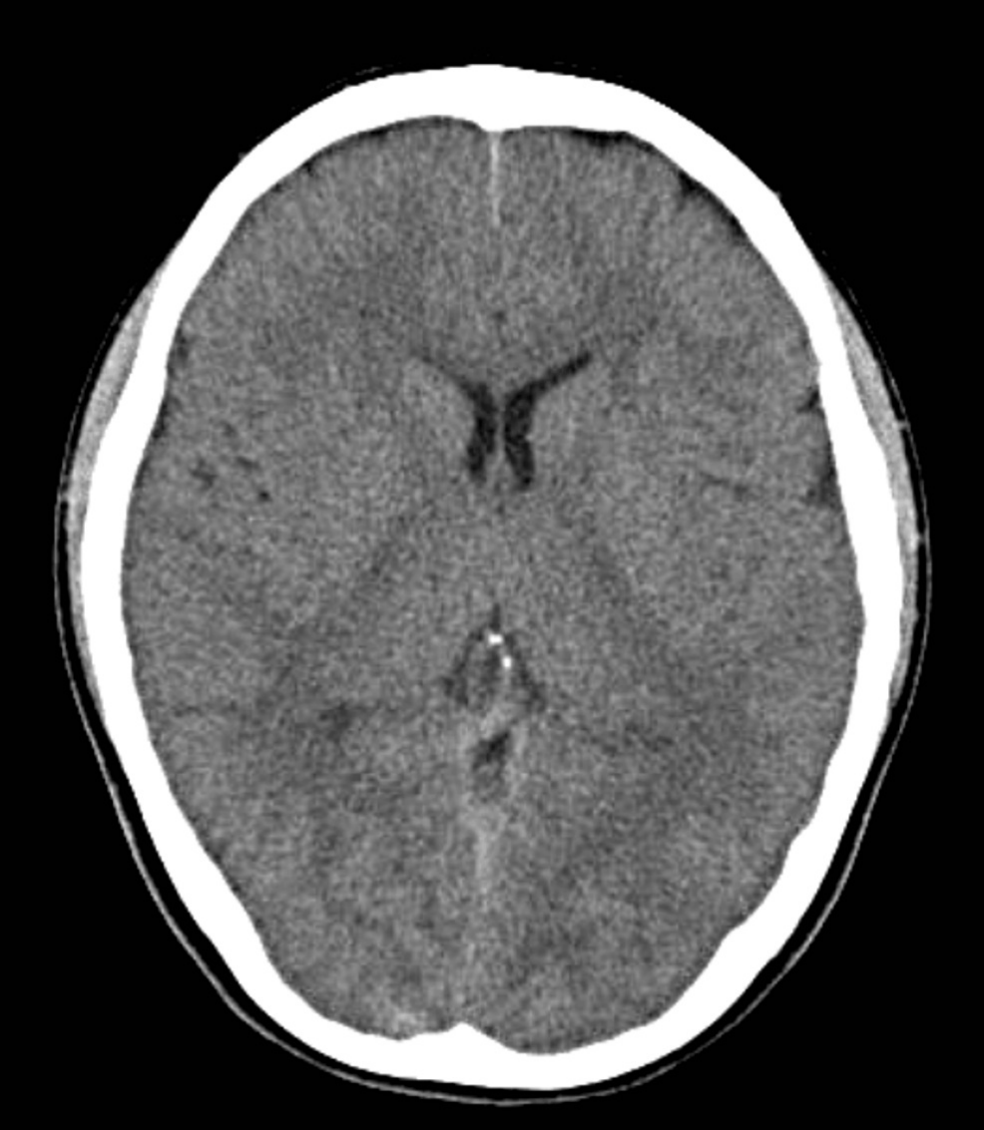
Head computed tomography performed on admission, showing an absence of calcification of the basal ganglia

Blood tests on admission (Table 1) showed marked hypocalcemia (adjusted serum calcium (Ca) level, 1.40 mmol/L; normal range: 2.20-2.52 mmol/L). Her ECG revealed QT prolongation (QTc = 0.524 s) (Figure 1A). Head computed tomography shows no calcification of the basal ganglia (Figure 2). Her hypocalcemia was corrected with 10 mL of intravenous 8.5% calcium gluconate, followed by oral calcium supplementation. Her serum Ca level increased to 1.63 mmol/L on day 2, and the QT interval on ECG (QTc) decreased to 0.465 s (Figure 1B). She was diagnosed with acquired QT prolongation due to hypocalcemia.

**Table 1:** The patient’s blood test results on admission.

Test	Reference range	Result
Hemoglobin (g/L)	119–149	138
White blood cell count (10^9^/L)	3.8–10.1	5.17
Differential (proportion of 1.0)		
Neutrophils	0.45–0.72	0.42
Eosinophils	0.008–0.084	0.046
Platelet count (10^9^/µL)	180–440	208
C-reactive protein (mg/L)	0–1.4	0.00
Albumin (g/L)	41–51	47
Globulin (g/L)	25–30	23
Total bilirubin (µmol/L)	6.84–25.66	8.55
Aspartate aminotransferase (U/L)	13–30	31
Alanine aminotransferase (U/L)	10–42	14
Alkaline phosphatase (U/L)	155–900	272
Creatinine kinase (U/L)	59–248	832
Urea nitrogen (mmol/L)	2.86–7.14	4.89
Creatinine (µmol/L)	35.36–61.88	43.32
Sodium (mmol/L)	138–145	141
Potassium (mmol/L)	3.6–4.8	3.6
Chloride (mmol/L)	101–108	104
Calcium (mmol/L)	2.20–2.52	1.40
Ionized calcium (mmol/L)	1.15–1.33	0.70
Phosphate (mmol/L)	0.87–1.49	2.65
Magnesium (mmol/L)	0.74–0.95	0.74
Glucose (mmol/L)	4.05–11.10	5.22
Thyroid stimulating hormone (mIU/L)	0.5–5.0	2.38
Free T3 (pmol/L)	3.54–6.62	5.41
Free T4 (pmol/L)	11.58–21.88	17.76
Intact parathyroid hormone (ng/L)	10–65	21
1α,25-dihydroxy vitamin D (pmol/L)	48.0–144.0	230.6
pH	7.35–7.45	7.38
Bicarbonate (mmol/L)	20.0–26.0	21.5
Base excess (mmol/L)	−3.0 to 3.0	−3.1
Lactate (mmol/L)	0.4–1.8	2.68

Hypoparathyroidism was suspected as the etiology of the hypocalcemia because the patient’s intact parathyroid hormone (PTH) level was not elevated (21 ng/L; normal range: 10-65 ng/L) despite the presence of hyperphosphatemia (serum phosphate, 2.7 mmol/L; normal range: 1.16-1.87 mmol/L) and hypocalcemia. Her serum magnesium level was within the normal range (0.74 mmol/L; normal range: 0.74-0.95 mmol/L). Hypercalciuria was excluded based on a urine calcium:creatinine ratio of 0.013 (normal range: <0.14).

**Figure 3:**
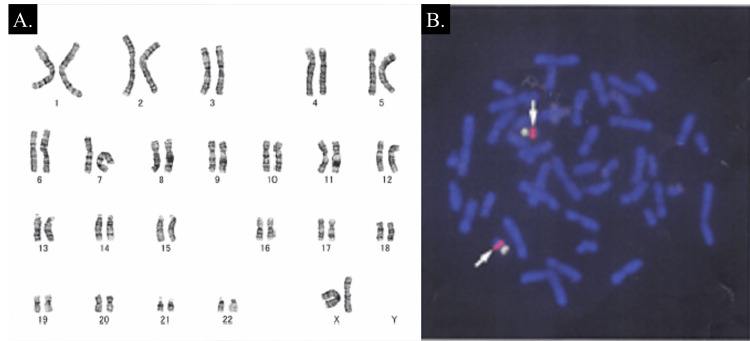
Genetic testing of the patient (A) Chromosome analysis showing a normal G-banded chromosome (46,XX). (B) Fluorescence in situ hybridization showing a lack of deletion or duplication of the domain of 22q11.2 responsible for 22q11.2 deletion syndrome (red with white arrows).

**Figure 4:**
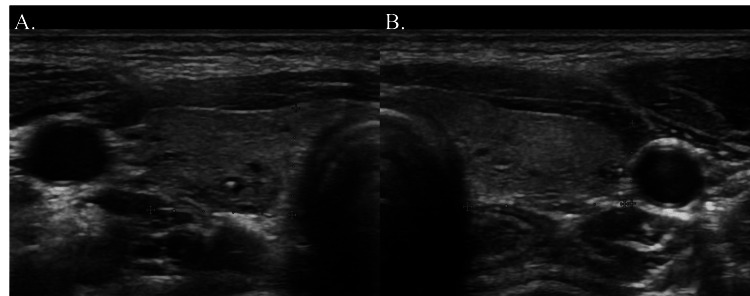
Ultrasound image of the thyroid of the patient (A) Right lobe of the thyroid. (B) Left lobe of the thyroid. The parathyroid glands were not observed.

Additional diagnostic tests to exclude DiGeorge syndrome type 1 as the etiology of the patient’s hypoparathyroidism showed normal G-banded chromosome analysis findings (46,XX) and no 22q11.2 deletion on fluorescence in situ hybridization screening (Figures 3A, 3B). There were no destructive lesions of the parathyroid gland on ultrasound (Figures 4A, 4B). No abnormalities were detected on audiometry, glucose tolerance, thyroid function, autoimmune tests, echocardiography, Holter ECG, or electroencephalography (EEG). DiGeorge syndrome type 1, other malformation syndromes, and the polyglandular autoimmune syndrome were ruled out on the basis of these test results. No further investigations were performed to investigate specific genetic causes.

Based on these findings, the patient was diagnosed with primary hypoparathyroidism. The patient’s serum Ca levels were controlled with supplementation of activated vitamin D (1-alfacalcidol) (3 µg/day) and Ca (390 mg/day). The trends in her serum Ca and PTH levels during the course of hospital admission are shown in Table 2. She has not experienced any recurrence of syncope over the subsequent two years.

**Table 2:** Treatment and trends in serum calcium and parathyroid hormone levels during hospital admission

	Reference range	Day 0	Day 1	Day 2	Day 3	Day 6	Day 8	Day 10	Day 18
Blood test results									
Calcium (mmol/L)	2.20–2.52	1.40	1.48	1.63	1.70	··	1.85	1.88	2.23
Intact parathyroid hormone (ng/L)	10–65	21.0	··	··	25.3	··	23.7	··	··
Supplements									
Calcium (mg/day)	··	235	390	390	390	390	390	390	260
Activated vitamin D_3_ (µg/day)	··	··	··	··	1	2	2	3	3

## Discussion

Idiopathic hypoparathyroidism is a rare endocrine disorder that can present at any age [3]. DiGeorge syndrome type 1 (22q11.2 deletion syndrome) is a common cause of pediatric hypoparathyroidism. This syndrome causes congenital heart disease, characteristic facial features, psychiatric illness, palatal dysfunction, thymic hypoplasia, and hypocalcemia [4]. Although 22q11.2 deletion syndrome and chromosomal abnormalities were ruled out in this case, a genetic etiology could not be ruled out as the patient was not evaluated for other genetic abnormalities such as *NEBL*, *TBCE*, *FAM111A*, *CASR*, and *GNA11* mutations [4]. However, acquired hypoparathyroidism due to causes other than neck surgery or radiation therapy is extremely rare [5], with the exception of hypomagnesemia, infiltrative diseases, such as Wilson’s disease and hemochromatosis, and thalassemia. Although the patient’s serum copper and ferritin were not checked, given the absence of other characteristics, such as psychological symptoms, jaundice, liver dysfunction, and microcytic anemia, the patient probably had congenital or idiopathic hypoparathyroidism.

Although we had no baseline serum Ca measurement, we assumed that the hypocalcemia had an acute onset because a routine ECG, performed during a periodic school health examination two months before the patient’s syncopal attack had been normal. However, the patient’s QT interval normalized on day two even before her hypocalcemia normalized, suggesting that asymptomatic hypocalcemia might have been present for a long time before developing the syncope. Nevertheless, the possibility that the hypocalcemia progressed rapidly and that the idiopathic hypoparathyroidism was of recent onset cannot be ruled out, given that head CT did not show calcification of the basal ganglia and that the patient presented with numbness on admission. A recent case report of primary hypoparathyroidism, possibly aggravated by COVID-19, concluded that long-term, subclinical hypocalcemia can cause cataracts at a young age and basal ganglia calcification [6]. Neurological manifestations appear to be less likely to occur if hypocalcemia is of gradual onset [5].

In this case, the patient developed sudden loss of consciousness associated with primary hypoparathyroidism, although we could not determine whether the loss of consciousness was due to a hypocalcemia-induced seizure or to non-convulsive syncope associated with ventricular arrhythmia caused by QT prolongation because no EEG or ECG was performed during the attack. Notably, hypoparathyroidism with loss of consciousness can be misdiagnosed as epilepsy or as a psychiatric disorder [7]. Therefore, it is important to consider the possibility of hypoparathyroidism or QT prolongation secondary to hypocalcemia, in patients with unexplained syncope or convulsions, and a workup should be performed to determine the etiology of abnormal ECG findings, even in adolescents.

Decreased Ca concentrations enhance neuronal and muscle excitability, resulting in facial and limb numbness, muscle cramping (carpopedal spasm), twitching (Chvostek’s sign and Trousseau’s sign), tetany, and generalized convulsions. Although a previous case report described generalized seizures associated with ventricular arrhythmias [8], severe hypocalcemia (serum Ca ≤0.84 mmol/L) without ECG abnormalities has been reported as a cause of tonic-clonic seizures [9].

It is crucial to perform ECG in patients presenting with loss of consciousness. In some cases, the cause of loss of consciousness can be identified using ECG alone, as this method is non-invasive and inexpensive and can reveal possible causes of syncope (such as Wolff-Parkinson-White syndrome and QT prolongation). Moreover, QT prolongation can produce ventricular arrhythmias such as torsades de pointes. In this case, the patient’s rapid awakening without convulsion suggested that she had experienced syncope associated with ventricular arrhythmia due to QT prolongation, although there was no direct evidence of cardiac syncope.

Although QT prolongation and ventricular arrhythmia are characteristic of hypocalcemia, there have been few reports of hypoparathyroidism causing ventricular arrhythmias. Jacob et al. recently reported a case of hypoparathyroidism in a 23-year-old woman secondary to hypomagnesemia [10]; this patient presented with sudden cardiac arrest due to torsades de pointes, though she had originally been suffering from rheumatic heart disease.

## Conclusions

In conclusion, hypoparathyroidism may be overlooked if symptoms related to hypocalcemia are absent. The etiology of transient loss of consciousness and ECG abnormalities should be explored, including serum electrolyte levels such as Ca. The cause of acquired QT prolongation should be investigated fully.
